# Genome-wide identification of endogenous viral sequences in alfalfa (*Medicago sativa* L.)

**DOI:** 10.1186/s12985-021-01650-9

**Published:** 2021-09-09

**Authors:** Alexander M. Boutanaev, Lev G. Nemchinov

**Affiliations:** 1 Institute of Basic Biological Problems, Russian Academy of Sciences, Pushchino, Moscow Region Russia; 2 USDA/ARS, Beltsville Agricultural Research Center, Molecular Plant Pathology Laboratory, Beltsville, MD 20705 USA

**Keywords:** Alfalfa, *Medicago sativa* L., Endogenous viral elements, Soybean chlorotic mottle virus, Figwort mosaic virus

## Abstract

Endogenous viral elements (EVEs) have been for the most part described in animals and to a less extent in plants. The endogenization was proposed to contribute toward evolution of living organisms via horizontal gene transfer of novel genetic material and resultant genetic diversity. During the last two decades, several full-length and fragmented EVEs of pararetroviral and non-retroviral nature have been identified in different plant genomes, both monocots and eudicots. Prior to this work, no EVEs have been reported in alfalfa (*Medicago sativa* L.), the most cultivated forage legume in the world. In this study, taking advantage of the most recent developments in the field of alfalfa research, we have assessed alfalfa genome on the presence of viral-related sequences. Our analysis revealed segmented EVEs resembling two dsDNA reverse-transcribing virus species: *Soybean chlorotic mottle virus* (family *Caulimoviridae*, genus *Soymovirus*) and *Figwort mosaic virus* (family *Caulimoviridae*, genus *Caulimovirus*). The EVEs appear to be stable constituents of the host genome and in that capacity could potentially acquire functional roles in alfalfa’s development and response to environmental stresses.

**Supplementary Information:**

The online version contains supplementary material available at 10.1186/s12985-021-01650-9.

## Introduction

Endogenous viral elements (EVEs) are partial or entire viral genes or genomes integrated into host chromosomes and inherited as alleles [1, 2]. EVEs may be eventually removed from the host genomes or reach fixation and retained for millions of years [1, 3, 4]. They are instrumental in a gene flow between viruses and eukaryotes and may have a substantial role in the evolution of their hosts [1, 5, 6]. While the majority of retroviral EVEs originate from a mandatory genome integration stage in the life cycle of reverse-transcribing viruses [1], insertion mechanisms of plant viruses is debated because integration of a DNA copy into the host genome is not required for their replication [7].

In plant genomes, two categories of EVEs are recognized: endogenous pararetroviruses (EPRVs), derived from the reverse-transcribing double stranded (ds)DNA viruses of the family *Caulimoviridae* and endogenous non-retroviral elements (ENREs), originating from dsRNA, ssRNA, and ssDNA viruses [4, 5, 7–9]. Several mechanisms for the integration of EPRVs into the plant genome were proposed, among them homologous and illegitimate recombination, integration through reverse-transcribed replication intermediates and via short AT-rich motifs flanking viral segments [8, 10, 11].

Insertion mechanisms of the ENREs remain largely uncharacterized and may involve reverse transcription by reverse transcriptase encoded by retrotransposons, recombination events, and transposon-mediated integration [5]. Localization and distribution of EVEs in plant genomes varies; it was reported that they might be located closer to transposable elements, overlap with genes [12], reside between the genes or within introns [5]. Numerous EVEs of both classes have been identified in the genomes of different plant species, including a close relative of alfalfa, *Medicago truncatula* [5, 7, 8, 12, 13].

To our knowledge, prior to this work, none were reported in the genome of alfalfa (*M. sativa*), widely cultivated perennial legume and important agricultural crop. In this study, taking advantage of the most recent developments in the field of alfalfa genomics, we performed a comprehensive, genome-wide screening aimed to identify virus-related sequences integrated into the alfalfa genome.

## Results

Two *M. sativa* genomes, tetraploid [14] and diploid (http://www.medicagohapmap.org/downloads/cadl), were used to create a standalone BLAST [15] database. At the time of this project’s initiation, the tetraploid genome was assembled to the chromosome level, while the diploid genomic sequences were available as scaffolds. To identify possible endogenous viral elements in the *M. sativa* genome we first used plant viral protein sequences from the Uniprot database (https://www.uniprot.org). These sequences were downloaded and piped through the *M. sativa* tetraploid and diploid genomes using the tBLASTn program. The standalone BLAST search was also used against reference viral database (https://www.ncbi.nlm.nih.gov/genome/viruses/).

The BLAST analyses revealed sequences resembling two plant virus species of the family *Caulimoviridae* and integrated into both tetraploid and diploid genomes of *M. sativa*: *Soybean chlorotic mottle virus* (SbCMV) of the genus *Soymovirus* and *Figwort mosaic virus* (FMV) of the genus *Caulimovirus*. The resultant parameters of the BLASTn output and the coordinates of the detected homology regions in the *M. sativa* genomes are shown in Table 1 and Additional file 2: Table S1. The two viral genomes (for Genbank accessions see Table 1) were further used in BLASTn search to confirm the location of the integrated viral sequences. All EVEs found in *M. sativa* tetraploid and diploid genomes are shown in the Additional file 1: File S1.

**Table 1:** EVEs identified in the *M. sativa* genome

Accession	Virus	Hits	Chr involved	Identity, %	Size, bp	Coverage of viral genome, %	Viral protein
***M. sativa*, tetraploid, chromosome assembly**							
NC_001739.2	AePV	1	1/32	75	241	12	Movement
NC_003554.1	FMV	41	24/32	82	87	1	capsid, putative
***M. sativa*, diploid, scaffold assembly**							
NC_001739.2	AePV	1	N/A	72	563	8	Movement
NC_003554.1	FMV	19	N/A	82	89	1	capsid, putative

The identity, size, and coverage are represented as the averages if the hit numbers are greater than 1. Proteins are named according to the Uniprot database

The SbCMV-like EPRVs were homologous to the genes encoding reverse transcriptase and movement protein (MP) of SbCMV. Since reverse transcriptase sequences were most probably derived from the multiple integrated transposons and could intervene with the analysis and identification of EPRVs, they were excluded from consideration. The SbCMV-like EPRVs found in both tetraploid and diploid genomes and corresponding to the MP overlapped (percent identity 90%). The top BLAST hit for the longest insert from the diploid genome (563 bp) was SbCMV isolate from Japan (X15828.2; 70%; query cover 100%; E-value = 2e−63). Translation of the SbCMV-like sequence resulted in 163 amino acid-long fragment that had 59% identity with SbCMV movement protein (NP_044299.1; query cover 100%; E-value = 4e−66). Low identity levels (more than 20% of both nucleotide and amino acid differences) indicated that this sequence could belong to a new pararetrovirus, which we provisionally named alfalfa endogenous pararetrovirus (AePV).

**Fig. 1:**
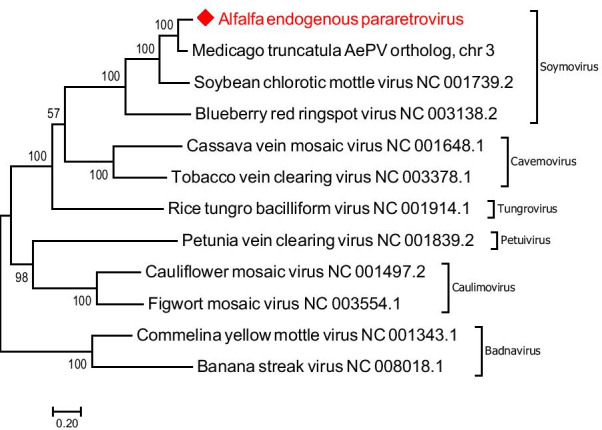
Phylogenetic relationship between AePV EPRVs and complete genomic sequences of the representative species from each genus of the family *Caulimoviridae*. The AePV EPRV designated with a red rhomb. Unrooted phylogenetic tree was deduced from the ClustalW alignment and built using MEGA 7 software with Maximum Likelihood method based on the Tamura-Nei model and bootstrap analysis of 1000 replicates

To confirm an identity of the discovered AePV EPRVs, we performed a phylogenetic analysis with the representative viruses. Phylogenetic trees were deduced from ClustalW alignments of the AePV sequence and the complete nucleotide sequences of the representative species from the family *Caulimoviridae* and built using MEGA 7 software [16] with Maximum Likelihood method based on the Tamura-Nei model and bootstrap analysis of 1000 replicates. Phylogenetic analysis clustered AePV together with SbCMV, thus confirming their relationship (Fig. 1). The group branched to another species in the genus *Soymovirus*, *Blueberry red ringspot virus*. All other members of the family grouped correspondingly to their respective genera.

Screening the AePV fragment against the genome of *Medicago truncatula*, a close relative of alfalfa, using the Plant Genomic Resource Phytozome 12 (https://phytozome.jgi.doe.gov/pz/portal.html), resulted in the identification of orthologous sequences located in all eight chromosomes of the species as well as in several scaffolds (Table 2). Representative orthologous sequence from the chromosome 3 of *M. truncatula* clustered together with the AePV, supporting their close relationship (Fig. 1).

**Table 2:** Orthologous EVEs found in the genome of *Medicago truncatula*

Chromosome	Chr coordinates, SbCMV	Chr coordinates, FMV-like
1	10486792..10486954	
2	37269632..37270250	32259959..32260049
3	25608192..25608810	
4	31133614..31134231	56466689..56466775
5	22051806..22052424	22429795..22429835
6	29624408..29625026	34120328..34120418
7	15686218..15686835	
8	37292977..37293610	

When the translated portion of the AePV or the orthologous sequence of *M. truncatula* were searched against the genome of *M. truncatula* (taxid 3880), a first BLASTp hit corresponded to the “viral movement protein” (AES67244.1) that was 97% identical (100% query cover; E-value = 3e-111) to the AePV. This indicates a presence of the AePV EPRVs in both species.

Short FMV-like EPRVs were found in most chromosomes of the tetraploid genome and scaffold assemblies of the diploid genome (Table 1). The sequences overlapped with each other, forming a 93 nt consensus sequence with a minimum/maximum percent match 85%/100% (Additional file 3: Figure S1). In BLASTn search optimized for high similarity, the consensus sequence had only one hit—with the FMV capsid protein (query cover 97%; E-value = 2e−09; identity 80.43%; X06166.1). When BLASTn was optimized to search for the somewhat similar sequences, the best hits also included portions of the capsid proteins of other species in the family *Caulimoviridae*.

**Fig. 2:**
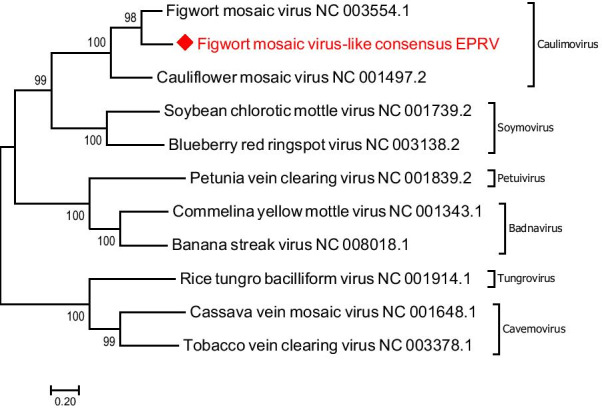
Phylogenetic relationship between the consensus sequence of the FMV-like EPRV and complete genomic sequences of the representative species from each genus of the family *Caulimoviridae*. The FMV-like EPRV designated with a red rhomb. Unrooted phylogenetic tree was deduced from the ClustalW alignment and built using MEGA 7 software with Maximum Likelihood method based on the Tamura-Nei model and bootstrap analysis of 10,000 replicates

Phylogenetic trees, deduced from the alignment of the FMV-like consensus sequence and complete genomes of the representative members of the family *Caulimoviridae*, placed FMV-like consensus in the same cluster with the reference sequence of figwort mosaic virus (NC_003554.1; Fig. 2). Thus, while the FMV-like EPRVs certainly belong to *Caulimoviridae* and are in all probability resembling FMV, the sequences are too short to make any definite conclusions on their exact identity. The search of the FMV-like consensus against genome of *M. truncatula* resulted in multiple hits (Table 2), implying that the EPRVs are present in the genome of this species as well.

**Fig. 3:**
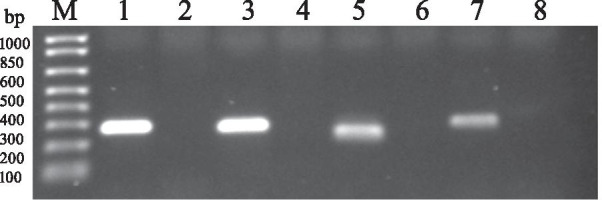
Confirmation of the randomly selected EVEs by PCR**.** M, 1 Kb Plus DNA Ladder (Thermo Fisher Scientific, USA); Lane 1: a 293 bp PCR product amplified using alfalfa DNA and primers pair LN968/969 designed for the FMV-like insert in Msat4n/chr1.1. Lane 2: amplification from *P. sativum* DNA using primers pair LN968/969. Lane 3: a 276 bp PCR product amplified from alfalfa DNA using primers pair LN970/971 for the FMV-like insert located in Msativa4n/chr2.2. Lane 4: Amplification from *P. sativum* DNA using primers pair LN970/971. Lane 5: a 219 bp PCR product amplified using alfalfa DNA and primers pair LN978/979 for the FMV-like insert in Msat/chr3.2. Lane 6: Amplification from *P. sativum* DNA with primers pair LN978/979. Lane 7: a 296 bp PCR product amplified using alfalfa DNA and primers pair LN980/981 for the FMV-like insert located in Msat/chr5.1. Lane 8: amplification from *P. sativum* DNA using primers pair LN980/981

To ensure that identified EPRVs are not artifacts or contaminants in sequence databases, they were randomly examined by PCR following by Sanger sequencing of the PCR products. DNA was extracted using DNeasy Plant kit (Qiagen) from one-week-old seedlings of alfalfa (*M*. *sativa* L) cv. Regency SY and common pea (*Pisum sativum*) cv. Lincoln, germinated in Petri dishes on moistened sterile filter paper. Prior to germination, alfalfa seeds were scarified with H_2_SO_4_, surface-sterilized with 70% ethanol for 1 min and rinsed with distilled water. PCR was performed using AmpliTaq Gold 360 DNA Polymerase under conditions recommended by the manufacturer (Thermo Fisher Scientific, USA), in two–three technical replicates. PCR primers used in the assays were specific to the alfalfa sequences located outside of the selected endogenous elements (Additional file 2: Table S1). DNA from common pea (*P*. *sativum* cv. Lincoln) served as a negative control. PCR products were purified with Qiagen PCR Purification kit and sequenced at Macrogen USA facility (Macrogen, MD USA). In all cases, PCR led to the amplification of the products of the expected size (Fig. 3). Sequencing of the purified reactions confirmed that they contained targeted regions incorporating EPRVs. These experiments verified computational findings.

## Discussion

In conclusion, we report the identification of two types of EPRVs in publicly available genomes of tetraploid and diploid alfalfa: SbCMV-like endogenous segments, presumably representing a novel virus in the family *Caulimoviridae*, tentatively named AePV; and FMV-like elements, which could not be classified assuredly due to the small size, although their homology to FMV and other members of the family *Caulimoviridae* is evident.

While great many EVEs have been found in the genomes of various plant species, as far as we know, none have been reported in alfalfa prior to this work [5, 7, 8, 12, 13]. This is likely due to the absence of the alfalfa genome, which only recently became available [14].

Pararetroviruses of the family *Caulimoviridae* identified in this study represent the most common group of EVEs in plants. They have been proposed to be major components of various plant genomes, possibly contributing to evolution of their hosts as sources of novel genetic material [12]. Despite that two viruses, SbCMV and FMV, which are similar to the EPRVs found in this study, have not currently been reported to infect alfalfa, these infections might occur in nature but so far had gone unnoticed. As a matter of fact, our unpublished results indicate that SbCMV and FMV are present among other viral genomes detected by high-throughput sequencing in field alfalfa samples (Nemchinov et al. unpublished).

Based on the BLASTn results and phylogenetic analysis, it can be assumed that the integration events recorded here are unlikely of recent nature and most probably occurred at some point during the species evolution. The diploid genome used in this work originated from CADL alfalfa (Cultivated Alfalfa at the Diploid Level), developed from cultivated tetraploids using haploidy, with breeding and selection [17]. It is, therefore, plausible that EPRVs identified in the CADL genome could be traced back to the tetraploid germplasm. This implies that they have reached fixation in the alfalfa genome, were inherited as alleles, and may presumably carry a functional load [12, 18].

Of two classes of EPRVs, only FMV-like sequences were found in most chromosomal sets of the tetraploid genome (Table 1). This may suggest, although speculatively due to the small size of the FMV inserts, that the integration events occurred before the whole genome duplication and both diploid progenitors of the tetraploid genome [19] contained EPRVs.

Hypothetically, uncovering the orthologous viral sequences in the genome of *M. truncatula* may further postpone the timetable of the integration events to at least ~ 5.3 million years ago, before alfalfa divergence from *M. truncatula* [14]. Therefore, it appears that EPRVs are stable constituents of the host genome. As a result, they could potentially acquire functional roles in alfalfa’s normal growth, organ development, metabolism, and response to environmental stresses [12]. It cannot be ruled out that EPRVs in alfalfa may also represent a source of infection.

## Supplementary Information

**Additional file 1**.** File S1**: Endogenous viral sequences identified in tetraploid and diploid genomes of M. sativa

**Additional file 2**.** Table S1**, page 1: Coordinates of the M. sativa genomic regions homologous to the SbCMV and FMV sequences.** Table S1**, page 2: PCR primers used for confirmation of randomly selected endogenous viral sequences

**Additional file 3**.** Figure S1**.** A**, a consensus sequence of the FMV-like EPRVs assembled using SeqMan tool of the DNASTAR software (DNASTAR, Inc. Madison, Wisconsin USA).** B**, a fragment of the DNASTAR alignment that generated a consensus sequence.

## Data Availability

Nucleotide sequences supporting reported results can be found in the Additional file 1: File S1.

## Abbreviations

EVEs: Endogenous viral elements
EPRVs: Endogenous pararetroviruses
ENREs: Endogenous non-retroviral elements
SbCMV: Soybean chlorotic mottle virus
FMV: Figwort mosaic virus
MP: Movement protein
AePV: Alfalfa endogenous pararetrovirus
CADL: Cultivated Alfalfa at the Diploid Level
