# Supplementary material for: Genome-wide identification of endogenous viral sequences in alfalfa (Medicago sativa L.)
Source: Virol J. 2021 Sep 9;18:185. doi: 10.1186/s12985-021-01650-9 (PMC8428138; doi:10.1186/s12985-021-01650-9)
Supplement: Supplementary file 1 — Additional file 1. File S1: Endogenous viral sequences identified in tetraploid and diploid genomes of M. sativa [file 12985_2021_1650_MOESM1_ESM.pdf]

File S1: Endogenous viral sequences identified in *M. sativa* tetraploid and diploid genomes

Viral sequences integrated in *Medicago sativa* tetraploid genome*
===

>Msativa4n|movement-chr3.2-1 12102273 12102514 93375939 (SbCMV)
TATAGATCTATCTAAATCTATATCTTTTAGGGATACTCATATTTGTAAATTTATATCAAATTTCATTTTT
CCATATTTTAGATTACATTTTCCTTTTGCAATTATTGCTTGTTTCTTATCTAGAATCCTAGAGTCTTCTA
AGGCTATTTCTAAAGGTGAATCAATACCTTTCATGAAGGTACTTTTAATTAGAATTTGAATGGTTGATAT
ATGAATATAACCAATCTTTCTTCTTTCGTTTT
>Msativa4n|capsid-chr1.1-1 24245905 24245998 82459472
AGAAGAATTCATGTCCCAAAGGTAAGAAAACATGCACATGTTGGTTGTGTCATGAAGAAGGACATTATGC
AAATGAGTGTCCTAAGAAGAAAAA
>Msativa4n|capsid-chr1.1-2 61882589 61882679 82459472
AAAGAATTCATGTCCCAAAGGTAAGAAAACATGCACATGTTGGCTGTGTCATGAAGAAGGACATTATGCA
AATGAGTGTCCTAAGAAGAAA
>Msativa4n|capsid-chr1.1-3 52399578 52399668 82459472
AAAGAATTCATGTCCTAAAGGCAAGAAAACATGCACAAGTTGGTTGTGTCATGAAGAAGGACATTATGCA
AATGAGTGTCCTAAGAAGAAA
>Msativa4n|capsid-chr1.2-1 79956260 79956350 86910131
AAAGAATTCATGTCCCAAAGGTAAGAAAACATGCACATGCTGGTTGTGTCATGAAGAAGGACATTATGCA
AATGAGTGTCCTAAGAAGAAA
>Msativa4n|capsid-chr1.2-2 53436684 53436774 86910131
AAAGAATTCATGTCCTAAAGGCAAGAAAACATGCACAAGTTGGTTGTGTCATGAAGAAGGACATTATGCA
AATGAGTGTCCTAAGAAGAAA
>Msativa4n|capsid-chr1.2-3 76498370 76498441 86910131
TTTCTTTTTGGGACACTCATTTGCATAATGTCCTTCTTCATTACACAACCAACATGTGCATGTTTTCTTT
CC
>Msativa4n|capsid-chr1.3-1 22851264 22851357 79881340
AGAAGAATTCATGTCCCAAAGGTAAGAAAACATGCACATGTTGGTTGTGTCATGAAGAAGGACATTATGC
AAATGAGTGTCCTAAGAAGAAAAA
>Msativa4n|capsid-chr1.3-2 31015440 31015530 79881340
AAAGAATTCATGCCCCAAAGGCAAGAAAACATGCACATGTTGGTTGTGTCATGAAGAAGGACATTATGCA
AATGAGTGTCCTAAAAAGAAA
>Msativa4n|capsid-chr1.3-3 50975822 50975912 79881340
TTTCTTCTTAGGACACTCATTTGCATAATGTCCTTCTTCATGACACAACCAACATGTGCATGTTTTCTTA
CCTTTGGGACATGAATTCTTT
>Msativa4n|capsid-chr1.3-4 70582004 70582075 79881340
TTTCTTTTTGGGACACTCATTTGCATAATGTCCTTCTTCATTACACAACCAACATGTGCATGTTTTCTTT
CC
>Msativa4n|capsid-chr1.3-5 70593676 70593747 79881340
TTTCTTTTTGGGACACTCATTTGCATAATGTCCTTCTTCATTACACAACCAACATGTGCATGTTTTCTTT
CC
>Msativa4n|capsid-chr1.3-6 70618639 70618710 79881340
TTTCTTTTTGGGACACTCATTTGCATAATGTCCTTCTTCATTACACAACCAACATGTGCATGTTTTCTTT
CC
>Msativa4n|capsid-chr1.4-1 24248476 24248569 88815615
AGAAGAATTCATGTCCCAAAGGTAAGAAAACATGCACATGTTGGTTGTGTCATGAAGAAGGACATTATGC
AAATGAGTGTCCTAAGAAGAAAAA
>Msativa4n|capsid-chr1.4-2 57162046 57162136 88815615
AAAGAATTCATGTCCTAAAGGCAAGAAAACATGCACAAGTTGGTTGTGTCATGAAGAAGGACATTATGCA

AATGAGTGTCCTAAGAAGAAA
>Msativa4n|capsid-chr2.2-1 66145004 66145094 74215936
AAAGAATTCATGTCCCAAAGGCAAGAAAACATGCACATGTTGGTTGTGTCATGAAGAAGGACATTATGCA
AATGAGTGTCCTAAAAAGAAA
>Msativa4n|capsid-chr3.2-1 86276468 86276548 93375939
TTTCTTTTTGGGACACTCATTTGCATAATGTCCTTCTTCATTGCACAACCAACATGTGCATGTTTTCTTA
CCTTTGGGACA
>Msativa4n|capsid-chr3.4-1 58412334 58412399 100414524
GGAAAGAAAACATGCACATGTTGGTTGTGTAATGAAGAAGGACATTATGCAAATGAGTGTCCCAAA
>Msativa4n|capsid-chr4.1-1 44420974 44421064 90245664
AAAGAATTCATGCCCTAAAGGCAAGAAAACATGCACATGTTGGTTGTGTCATGAAGAAGGACATTATGCA
AATGAGTGTCCCAAAAAGAAA
>Msativa4n|capsid-chr4.2-1 48658594 48658684 93947428
AAAGAATTCATGCCCTAAAGGCAAGAAAACATGCACATGTTGGTTGTGTCATGAAGAAGGACATTATGCA
AATGAGTGTCCCAAAAAGAAA
>Msativa4n|capsid-chr4.3-1 76987568 76987658 90228617
AAAGAATTCATGTCCCAAAGGCAAGAAAAAATGTACATGTTGGTTGTGTCATGAAGAAGGACATTATGCA
AATGAGTGTCCCAAGAGGAAA
>Msativa4n|capsid-chr4.4-1 48637844 48637934 90896203
AAAGAATTCATGCCCTAAAGGCAAGAAAACATGCACATGTTGGTTGTGTCATGAAGAAGGACATTATGCA
AATGAGTGTCCCAAAAAGAAA
>Msativa4n|capsid-chr5.1-1 4026950 4027040 81211777
AAAGAATTCATGTCCCAAAGGTAAGAAAACATGCACATGTTGGCTATGCCATGAAGAAGGACATTATGCA
AATGAGTGTCCTAAGAAGAAA
>Msativa4n|capsid-chr5.2-1 79168509 79168599 84165483
AAAGAATTCATGTCCCAAAGGTAAGAAAACATGCACATGTTGGCTGTGTCATGAAGAAGGACATTATGCA
AATGAGTGTCCTAAGAAGAAA
>Msativa4n|capsid-chr5.3-1 12480248 12480338 80712490
AAAGAATTCATGTCCCAAAGGTAAGAAAACATGCACATGTTGGCTGTGTCATGAAGAAGGACATTATGCA
AATGAGTGTCCTAAGAAGAAA
>Msativa4n|capsid-chr5.4-1 66457654 66457747 78626892
TTTTTCTTCTTAGGACACTCATTTGCATAATGTCCTTCTTCATGACACAACCAACATGTGCATGTTTTCT
TACCTTTGGGACATGAATTCTTCT
>Msativa4n|capsid-chr5.4-2 53029604 53029674 78626892
TTTCTTTTTGGGACACTCATTTGCATAATGTCCTTCTTCATTACACAACCAACATCATGTTTTCTTTCCT
T
>Msativa4n|capsid-chr6.1-1 53224485 53224576 80303593
CTTTTTCTTCGGACACTCATTTGCATAATGTCCTTCTTCATGGCACAACCAACATGTGCAAGTTTTCTTT
CCTTTTGGGCAAGAATTTTTCT
>Msativa4n|capsid-chr6.2-1 71199572 71199661 89579199
TTCTTTTTGGGACACTCATTTGCATAATGTCCTTCTTCATTACACAACCAACATGTGCATGTTTTCTTTC
CTTTAGGGCATGAATTCTTT
>Msativa4n|capsid-chr6.4-1 39640845 39640936 64534737
CTTTTTCTTCGGACACTCATTTGCATAATGTCCTTCTTCATGGCACAACCAACATGTGCAAGTTTTCTTT
CCTTTTGGGCAAGAATTTTTCT
>Msativa4n|capsid-chr6.4-2 37908281 37908370 64534737
TTTCTTTTTAGGACACTCATTTGCATAATGTCCTTCTTCATGACACAACCAACATGTGCATGTTTTCTTG
CCTTTGGGGCATGAATTTTT
>Msativa4n|capsid-chr7.2-1 6578532 6578622 93528358
TTTCTTCTTAGGACACTCATTTGCATAATGTCCTTCTTCATGACACAACCAACATGTGCATGTTTTCTTG
CCTTTGGGACATGAATTCTTT
>Msativa4n|capsid-chr7.2-2 74367041 74367131 93528358

TTTCTTTTTGGGACACTCATTTGCATAATGTCCTTCTTCATTACACAACCAACATGTGCATGTTTTCTTA
CCTTTAGGGCATGAATTCTTT
>Msativa4n|capsid-chr7.2-3 80890790 80890880 93528358
AAAGAATTCATGTCCCAAAGGCAAGAAAACATGCACATGTTGGTTGTGTCATGAAGAAGGACATTATGCA
AATGAGTGTCCTAAGAAGAAA
>Msativa4n|capsid-chr7.3-1 20541108 20541198 91580142
CTTTTTTTTGGGACACTCATTTGCATAATGTCCTTCTTCATTACACAACCAACATGTGCATGTTTTCTTT
CCTTTAGGGCATGAATTCTTT
>Msativa4n|capsid-chr7.3-2 74966962 74967052 91580142
TTTCTTTTTGGGACACTCATTTGCATAATGTCCTTCTTCATTACACAACCAACATGTGCATGTTTTCTTA
CCTTTAGGGCATGAATTCTTT
>Msativa4n|capsid-chr7.4-1 30505904 30505995 94657719
AGAAAAATTCTTGTCCAAAAGGAAAGAAAACTTGCACATGTTGGTTGTGCCATGAAGAAGGACATTATGC
AAATGAGTGTCCTAAGAAAAAG
Msativa4n|capsid-chr8.1-1 45563162 45563252 87242343
TTTCTTCTTAGGACACTCATTTGCATAATGTCCTTCTTCATGACACAACCAACATGTGCATGTTTTCTTG
CCTTTGGGACATGAATTCTTT
>Msativa4n|capsid-chr8.1-2 38416637 38416728 87242343
CTTTCTTCTTATGACACTCATTTGCATAATGTCCTTCTTCATGACACAGCCAACATGTGCATGTTTTCTT
ACCTTTGGGACATGAATTCTTT
>Msativa4n|capsid-chr8.1-3 17091889 17091974 87242343
TTTCTTCTTAGGACACTCATTTGCATAATGTCCTTCTTCATGACACAACCAACATGTGCATGTTTTCTTA
CCTTTGGGACATGAAT
>Msativa4n|capsid-chr8.2-1 36532300 36532391 84274390
CTTTCTTCTTATGACACTCATTTGCATAATGTCCTTCTTCATGACACAGCCAACATGTGCATGTTTTCTT
ACCTTTGGGACATGAATTCTTT
>Msativa4n|capsid-chr8.3-1 42563286 42563376 82440740
TTTCTTCTTAGGACACTCATTTGCATAATGTCCTTCTTCATGACACAACCAACATGTGCATGTTTTCTTG
CCTTTGGGACATGAATTCTTT
==
=====================

Viral sequences integrated in *Medicago sativa* diploid genome*
==

>Msativa2n|movement-scaffold1037 349115 349677 504721 (SbCMV)
AAGAAAATTTTAAAAGAAAATTTAGTAAACTTAAGGAATTTCTGTCAAGAAAAAATATTCTAAAATTTGG
AATAATGGTAGGAGAGGAAGCAATTCCAATTGAAACAACTAATGGGAAAATAGTTATATCAACTATAGAT
AAACAACAAATCGATAGAAGACTTGAAAAATTTTCTGAAAATGAAAGAAGAAAGATTGGTTATATTCATA
TATCAACCATTCAAATTCTAATCAAAAGTACCTTCATGAAAGGTATTGATTCACCTTTAAAAATAGCTCT
AGAGGATTCTAGAATTAGAAATAAGAAACAAGCTGTAATTGCAAAAGGAAAATGTAATCTAAAATATGGA
CAGATAAAATTTGACATAAATTTACAAATGGGATTATCCCTAAAAGATATAGATCTGAATAAATCTATAG
TTTTTCATTATAAATTGGAAGACTCAACATTTATGAAAGAAGGTAATCATCCTTTTACAATTTACTACAG
AATTAACTATGCTTTGAGTAATTCACATCATAGCATAGAATTCATAGGAAAAGATACAATTCATATTGAT
GAA
>Msativa2n|capsid-scaffold1 1601229 1601319 1826351
TTTTTCGCATTGATTTCGGATCCTTCTGACTTTTTAAAGTACGTGTGTTGAATTTTGTTATTTTCTCTTT
TCAATATTTCAAATGTTTGAT
>Msativa2n|capsid-scaffold2341 61974 62064 112209
TTTCTTTTTAGGACACTCATTTGCATAATGTCCTTCTTCATGACACAACCAACATGTGCATGTTTTCTTG
CCTTTGGGGCATGAATTCTTT
>Msativa2n|capsid-scaffold356 215793 215884 739378

```
CTTTTTCTTTGGACACTCATTTGCATAATGTCCTTTTTCACGACATAACCAACATGTGCATGTTTTCTTT
CCTGTTGGACAGGAAGTTTTCT
>Msativa2n|capsid-scaffold1050 251233 251323 296422
TTTCTTCTTAGGACACTCATTTGCATAATGTCCTTCTTCATGACACAACAAACACGTGCATGTCTTCTTG
CCTTTAGGACATGAATTCTTT
>Msativa2n|capsid-scaffold24 679399 679489 1172671
AAAGAATTCATGTCCCAAAGGTAAGAAAACATGCACATGTTGGCTGTGTCATGAAGAAGGACATTATGCA
AATGAGTGTCCTAAGAAGAAA
>Msativa2n|capsid-scaffold537 814871 814961 1029455
TTTCTTCTTAGGACACTCATTTGCATAATGTCCTTCTTCATGACACAGCCAACATGTGCATGTTTTCTTA
CCTTTGGGACATGAATTCTTT
>Msativa2n|capsid-scaffold2519 99093 99182 157492
AAAGAATTCATGCCCTAAAGGAAAGAAAACATGCACATGTTGGTTGTGTAATGAAGAAGGACATTATGCA
AATGAGTGTCCCAAAAAGAA
>Msativa2n|capsid-scaffold342 808321 808411 981572
TTTCTTCTTAGGACACTCATTTGCATAATGTCCTTCTTCATGACACAACCAACATGTGCATGTTTTCTTG
CCTTTAGGACATGAATTCTTT
>Msativa2n|capsid-scaffold34 1556827 1556917 1744250
CTTTTTTTTGGGACACTCATTTGCATAATGTCCTTCTTCATTACACAACCAACATGTGCATGTTTTCTTT
CCTTTAGGGCATGAATTCTTT
>Msativa2n|capsid-scaffold657 124647 124737 524074
AAAGAATTCATGCCCTAAAGGTAAGAAAACATGCACATGTTGGTTGTGTAATGAAGAAGGACATTATGCA
AATGAGTGTCCCAAAAAGAAA
>Msativa2n|capsid-scaffold268 544456 544547 620361
AGAAAAAATTCTTGCCCAAAAGGAAAGAAAACTTGCACATGTTGGTTGTGCCATGAAGAAGGACATTATGC
AAATGAGTGTCCTAAGAAAAAG
>Msativa2n|capsid-scaffold396 266735 266806 543988
GGAAAGAAAACATGCACATGTTGGTTGTGTAATGAAGAAGGACATTATGCAAATGAGTGTCCCAAAAAGA
AA
>Msativa2n|capsid-scaffold3656 6129 6219 29304
aaagaattcatgccctaaaggtaagaaaacatgcacatgttggttgttgtaatgaagaaggacattatgc
aaatgagtgtcccaaaaagaa
>Msativa2n|capsid-scaffold1797 136864 136954 213233
TTTCTTTTTGGGACACTCATTTGCATAATGTCCTTCTTCATTACACAACCAACATGTGCATGTTTTCTTA
CCTTTAGGGCATGAATTCTTT
>Msativa2n|capsid-scaffold2114 44938 45023 136287
ATTCATGTCCCAAAGGTAAGAAAACATGCACATGTTGGTTGTGTCATGAAGAAGGACATTATGCAAATGA
GTGTCCTAAGAAGAAA
>Msativa2n|capsid-scaffold508 235157 235247 938428
TTTCTTCTTAGGACACTCATTTGCATAATGTCCTTCTTCATGACACAACCAACATGTGCATGTTTTCTTG
CCTTTGGGACATGAATTCTTT
>Msativa2n|capsid-scaffold377 767918 768009 1079537
CTTTTTCTTCGGACACTCATTTGCATAATGTCCTTCTTCATGGCACAACCAACATGTGCCAGTTTTCTTT
CCTTTTGGGCAAGAATTTTTCT
>Msativa2n|capsid-scaffold945 216128 216218 335058
AAAGAATTCATGTCCTAAAGGCAAAAAAACATGCACATGTTGGTTGTGTCATGAAGAAGGACATTATGCA
AATGAGTGTCCTAAGAAGAAA
>Msativa2n|capsid-scaffold1776 130122 130212 174602
AAAGAATTCATGCCCTAAAGGCAAGAAAACATGCACATGTTGGTTGTGTCATGAAGAAGGACATTATGCA
AATGAGTGTCCCAAAAAGAAA
======================================================================
===========================
```

NOTE: the last three numbers stand for sequence start, end, and chr/scaffold length, respectively.*All capsid EVEs belong to *Figwort mosaic virus*
