# Supplementary figures and images for: Genome-wide identification of endogenous viral sequences in alfalfa (Medicago sativa L.)
Source: Virol J. 2021 Sep 9;18:185. doi: 10.1186/s12985-021-01650-9 (PMC8428138; doi:10.1186/s12985-021-01650-9)

Figure S1

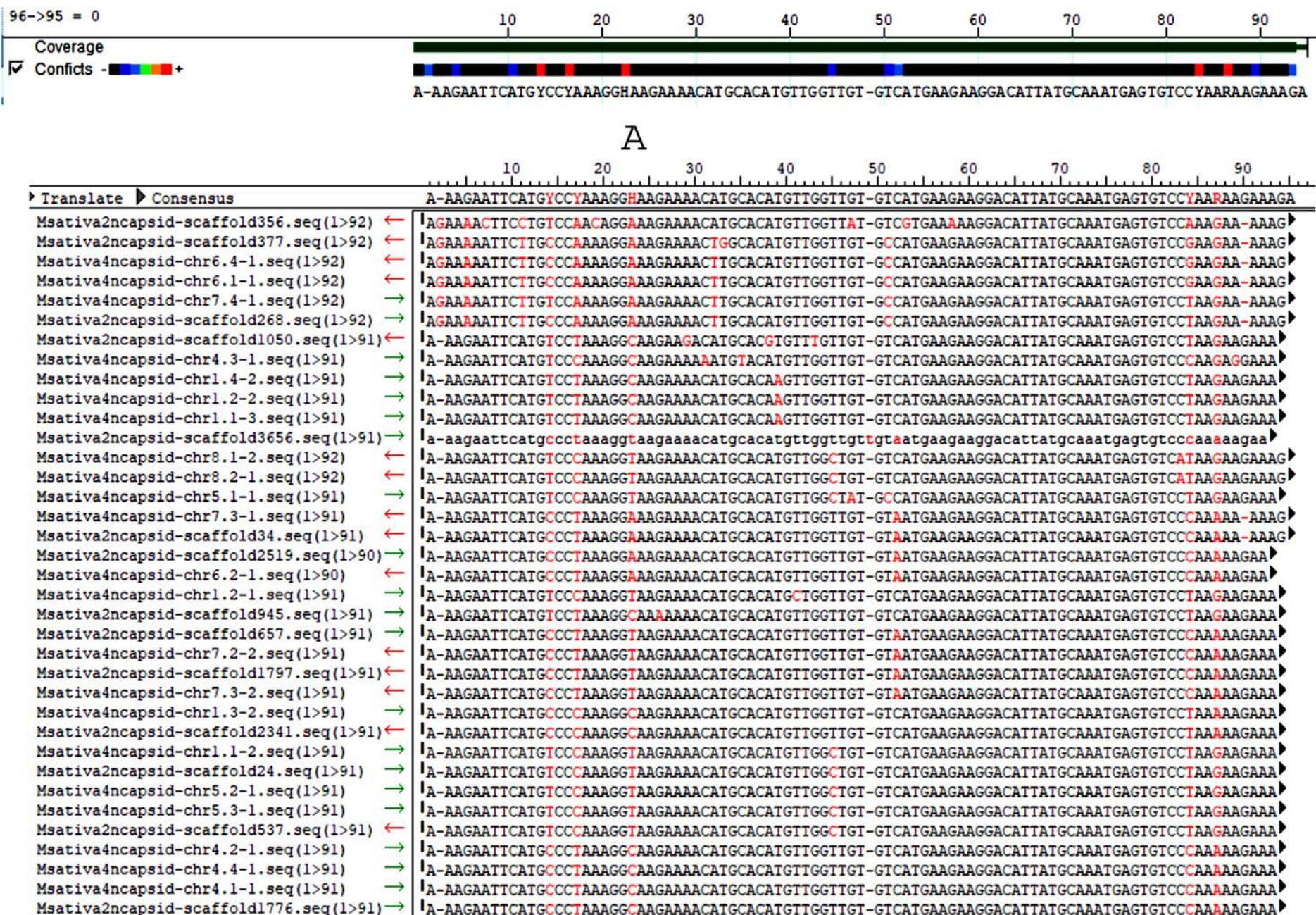

B

Supplement: Supplementary file 3 — Additional file 3. Figure S1. A, a consensus sequence of the FMV-like EPRVs assembled using SeqMan tool of the DNASTAR software (DNASTAR, Inc. Madison, Wisconsin USA). B, a fragment of the DNASTAR alignment that generated a consensus sequence. [file 12985_2021_1650_MOESM3_ESM.pdf]
